# Using tDCS as an Add-On Treatment Prior to FES Therapy in Improving Upper Limb Function in Severe Chronic Stroke Patients: A Randomized Controlled Study

**DOI:** 10.3389/fnhum.2018.00233

**Published:** 2018-06-19

**Authors:** Nuerjiayi Shaheiwola, Bin Zhang, Jie Jia, Dingguo Zhang

**Affiliations:** ^1^State Key Laboratory of Mechanical System and Vibration, Robotics Institute, Shanghai Jiao Tong University, Shanghai, China; ^2^Department of Rehabilitation, Huashan Hospital, Fudan University, Shanghai, China

**Keywords:** transcranial direct current stimulation, functional electrical stimulation, homeostatic mechanism, upper limb rehabilitation, stroke

## Abstract

**Background:** Upper limb function recovery is of vital importance for stroke patients. However, it is difficult to get ideal recovery, especially for patients with severe chronic stroke. As the first randomized controlled long-term trial combining bilateral transcranial direct current stimulation (tDCS) and functional electrical stimulation (FES) therapy, this study examined the efficacy of a novel protocol that included applying tDCS as an add-on treatment prior to FES therapy over the course of a 4-week program.

**Methods:** Thirty subjects with severe chronic stroke were randomized to either Group A (active tDCS+FES) (*N* = 15) or Group B (sham tDCS+FES) (*N* = 15). Five assessments including 3 behavioral outcome measurement scales [the Fugl-Meyer scale (cFMA), the Wolf motor function test (WMFT) and the modified Ashworth scale (MAS)], the surface electromyography (sEMG) evaluation and the transcranial magnetic stimulation (TMS) assessment were performed to evaluate subjects before and after the overall therapy.

**Results:** In Group A, the combined protocol was well tolerated by all patients and induced significant improvements in upper extremity motor abilities in terms of the assessments of cFMA [*t*_(14)_ = −5.658, *p* < 0.05], WMFT [*t*_(14)_ = −3.746, *p* < 0.05], MAS [*t*_(14)_ = 5.236, *p* < 0.05], sEMG and TMS. The results of between-group comparisons showed there was a significant difference between Group A and Group B in terms of the assessments of cFMA [*t*_(28)_ = 2.223, *p* < 0.05], WMFT [*t*_(28)_ = −2.152, *p* < 0.05] and sEMG [*F*_(1, 196)_ = 0.918, *p* < 0.05].

**Conclusion:** The proposed protocol can facilitate improvements in upper extremity motor abilities in severe chronic stroke patients and is more beneficial than the protocol with FES therapy alone. Our results showed efficacy of the new paradigm with combined intervention in both the central nervous system and the peripheral nervous system.

Trial registration: ChiCTR-ICR-15006108

## 1. Introduction

As one of the most devastating neurological conditions, stroke results in approximately 5.5 million deaths annually worldwide (Prentice et al., 2004). The worldwide prevalence of stroke survivors was estimated to be 62 million in 2005 and is predicted to reach 77 million by 2030 (Strong et al., 2007). About 65% of the patients experience upper-limb function impairment after 6 months of the stroke onset (Dobkin, 2005). Upper limb function accounts for approximately 60% of the whole body function, indicating that the self-managing ability and independence of patients after stroke mainly depends on upper limb recovery levels (Veerbeek et al., 2011). However, limb motor function recovery usually reaches a plateau 6 months after the onset of the stroke (Stinear, 2010; Stinear et al., 2012). Therefore, it is important to choose effective evidence-based interventions to further promote the recovery of hand function of chronic stroke patients.

Functional electrical stimulation (FES) is a well-studied technique that incorporates electrical stimulation to peripheral sensory and motor nerves with repetitive functional movement of the affected arm (Plonsey and Barr, 2000). Studies that used FES therapy reported significant improvements in upper limb functions of both chronic and acute stroke patients (Popovic et al., 2004, 2005). However, even with the help of regular FES therapy, acute and subacute stroke patients still suffer from a high risk of the upper limb function loss. Central to this problem is the lack of complimentary therapies that can better assist stroke patients in upper limb function recovery.

There is another non-invasive technique called transcranial direct current stimulation (tDCS) which may shed light on the problem (Bindman et al., 1964). The technique is able to induce sustained cortical excitability changes and modify cortical plasticity in the human cortex by shifting the resting potential of neuronal membranes (Nitsche and Paulus, 2001). Anodal tDCS facilitates motor-evoked potentials (MEPs) (Nitsche and Paulus, 2000, 2001) while cathodal tDCS inhibits them in contrast (Nitsche et al., 2003). Related studies have demonstrated the potential effects of tDCS on motor performance of stroke subjects (Fregni et al., 2005; Hummel et al., 2005; Boggio et al., 2007; Hesse et al., 2011). Particularly, cathodal stimulation is used to inhibit the non-lesioned hemisphere as the non-lesioned hemisphere partially overrides the lesioned hemisphere as a result of disturbed interhemispheric competition following stroke (Perez and Cohen, 2009). One study comparing different tDCS electrode montages on stroke subjects showed patients who responded to unilateral tDCS also responded to bilateral motor cortex stimulation, however, the effect of bilateral tDCS (with anodal stimulation in the affected hemisphere vs. cathodal stimulation in the unaffected hemisphere) might differ across patients and stroke characteristics (Mahmoudi et al., 2011).

Since both tDCS and FES therapy have been regarded as promising techniques for stroke rehabilitation, a combination of them may induce significant improvements compared with either one alone. There is some research on simultaneous application of non-invasive cortex stimulation and neuromuscular electrical stimulation on healthy subjects and stroke patients (Rosenkranz et al., 2000; Nitsche et al., 2007; Schabrun et al., 2013). One study evaluating motor task performance of subjects with chronic stroke revealed that combining peripheral nerve stimulation with anodal brain polarization induced superior improvements in performance of a motor task relative to the use of each intervention alone in combination with sham stimulation and training (Celnik et al., 2009). In terms of the combination of tDCS and FES therapy, one recent study by Menezes et al. (2017) assessed the short-term effect of different combinations of tDCS and repetitive peripheral nerve sensory stimulation (RPSS) as add-on treatments to the FES therapy in single sessions (Menezes et al., 2017). However, the long-term efficacy of combining both therapies, to our knowledge, has never been investigated.

In this research, we aimed to examine the long-term efficacy of this combined protocol over upper extremity motor abilities in severe chronic stroke patients. The bilateral montage was used in tDCS intervention. Our hypothesis was that the protocol of combining tDCS as an add-on treatment prior to the FES therapy would be superior to the FES therapy alone in the recovery of upper extremity motor abilities.

## 2. Methods

**Figure 1:**
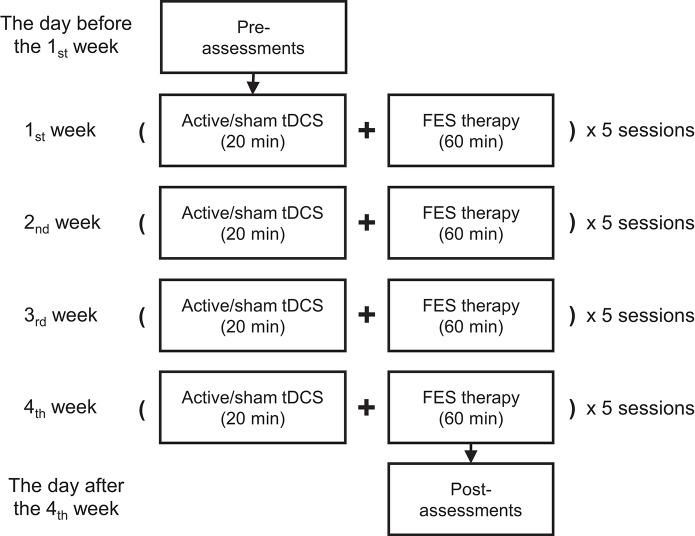
The proposed protocol with 20 sessions involving both tDCS and FES therapy over 4 weeks. Each session was carried out on a workday during the intervention period.

The ongoing comprehensive rehabilitation program consisted of 20 min of tDCS intervention followed by 1 h of FES therapy, altogether 80 min as a complete session for each day (see Figure 1). Five sessions per week on workdays and a total of 20 sessions were carried out during the 4 weeks of the intervention period. This study was approved by both the Chinese Clinical Trial Registry (registration No.: ChiCTR-ICR-15006108, date: 2015-03-15) and the Institutional Review Board of Huashan Hospital affiliated to Fudan University (approved No. of ethic committee: 2014 clinical trial No.279). The experiment was strictly performed in accordance with all relevant guidelines and regulations of the institutional review board.

### 2.1. Subjects

**Figure 2:**
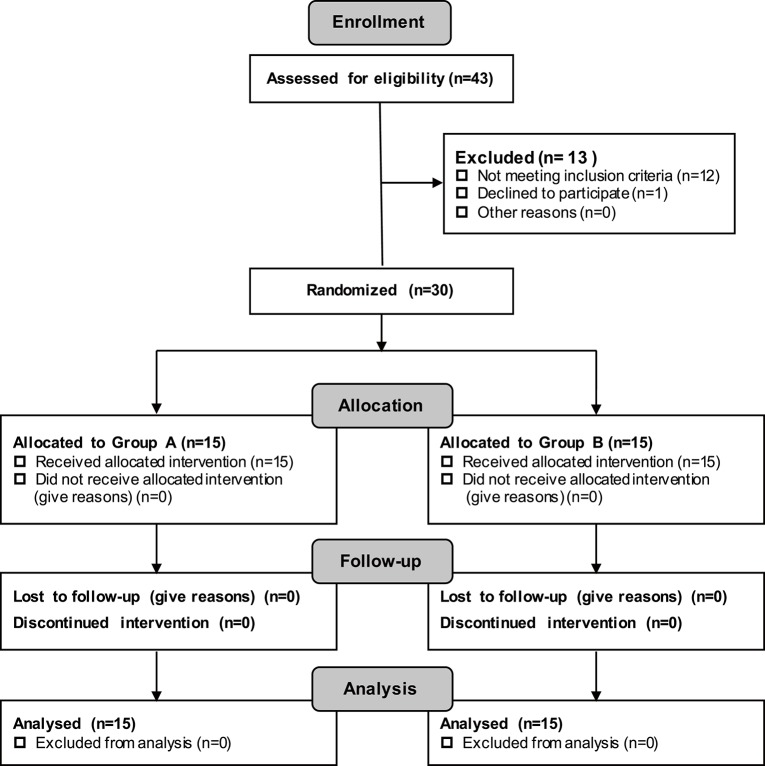
CONSORT Diagram Demonstrating the Recruitment Process and the Recruitment Numbers.

Patients were recruited via the official web platform of Huashan Hospital affiliated with Fudan University (Excluded *N* = 13; Not meeting the inclusion criteria *N* = 12; Declined to participate *N* = 1) (see Figure 2). Thirty subjects in total were recruited. All of the patients were diagnosed with stroke by a qualified physician before participating in the experiment. In this research, patients were classified as severe only if they satisfied the requirement of Brunnstorm recovery at Stage 0–3 in the paretic arm.

The inclusion criteria for this study were: (1) age between 35 and 70 years; (2) cerebral hemorrhage or cerebral infraction for the first time; (3) confirmed by head CT or MRI; (4) at least 6 months since stroke onset and an ipsilateral arm Brunnstrom recovery at stages 0–3; (5) conscious and able to communicate; and (6) able to sign informed consent himself/herself or with the help of his/her immediate family member.

The exclusion criteria were: (1) sequelae after lacunar cerebral infraction; (2) peripheral neuropathy in upper limbs; (3) unconsciousness, sensory aphasia or mental disorders, that may lead to failures in coordinating examination and treatment; (4) history of seizure. (5) serious illnesses, such as heart, liver or kidney diseases, or serious coagulation disorders; (6) history of cognitive disorder, neuropsychiatric disorder, drug or alcohol abuse; (7) organ failure, carcinoma or terminal stroke that seriously affect quality of life beyond hand dysfunction; (8) inability to complete basic course, to persist treatment, or difficult to follow-up; (9) with metal implants or skull defect; (10) existence of skin rash, allergy or wounds at the locations where stimulation electrodes would be placed.

### 2.2. Study protocol and randomization

All 30 recruited participants provided written informed consent and were then assigned randomly to either Group A (with active tDCS+FES therapy) or Group B (with sham tDCS+FES therapy) (see Table 1). Statistical results showed no significant baseline differences between Group A (active tDCS+FES therapy group) and Group B (sham tDCS+FES therapy group) in terms of age [*t*_(28)_ = −0.696, *p* = 0.492], gender (*p* = 1.000), months post-stroke onset (*Z* = −0.104, *p* = 0.917) and affected arm side (*p* = 0.715).

**Table 1:** Baseline data comparisons between Group A and Group B.

**Feature**	**Group A**	**Group B**	***p*-value**
Age, yr [Mean (SD)]	49.3 (9.4)	51.9 (11.0)	0.492
Gender, M (male) /F (female)	14 M/1 F	13 M/2 F	1.000
Since stoke, months [Median (IQR)]	18.0(15.0)	16.0(13.0)	0.917
Affected arm side, L (left) /R (right)	8L/7R	6L/9R	0.715
cFMA score [Mean (SD)]	15.9 (9.02)	18.2 (12.5)	0.564
WMFT score [Mean (SD)]	1.49 (0.34)	1.56 (0.58)	0.668
MAS score [Mean (SD)]	5.56 (1.82)	5.08 (1.52)	0.439

To ensure the reliability of results, there was a baseline observation period of 4 weeks before the intervention period. No intervention was performed throughout the baseline observation period. A blinded clinical rater assessed the upper limb function of all subjects before and after the baseline observation period. A preliminary analysis on baseline data (i.e., data before the baseline observation period) showed, cFMA scores [*t*_(28)_ = −0.585, *p* = 0.564], WMFT scores [*t*_(28)_ = −0.430, *p* = 0.668] and MAS scores [*t*_(28)_ = 0.786,*p* = 0.439] did not reach statistical significance between Group A and Group B (see Table 1). Additionally, no significant change was found in the baseline period in either group as regarding the results of the three chosen behavioral outcome measures.

### 2.3. Interventions

**(a) Transcranial direct current stimulation**. In tDCS application, a bilateral montage was chosen. The technique of tDCS was implemented with a commercial device (BrainStim stimulator, E.M.S. s.r.l., Italy). The subject was seated comfortably on a chair during the tDCS intervention. The APB (abductor pollicis brevis) hot spot for tDCS intervention was located by stimulating the primary motor cortex using TMS (MagPro R30 with MagOption, MagVenture, Denmark). TMS single-pulses with the maximum output intensity of 4.2 T were delivered via a figure-of-eight coil while the subject seated himself/herself with arms rested on their legs. The stimulation site on the scalp that produced the largest and the most consistent MEPs on the lesioned hemisphere was marked and considered as the hot spot for APB of the paralyzed arm. We placed the anode electrode (5 cm × 5 cm) of tDCS over the hot spot on the lesioned hemisphere and the cathode electrode (5 cm × 5 cm) on the contralateral symmetrical area of non-lesioned hemisphere. If MEPs could not be detected on the paralyzed arm, the hot spot for APB on the non-lesioned hemisphere was first determined, and then the symmetrical area on the contralateral hemisphere was regarded as the APB hot spot corresponding to the paralyzed arm. tDCS intervention was delivered for 20 min via a pair of sponge electrodes moistened with 0.9% NaCl solution. The active tDCS protocol (intensity: 2.0 mA, time of ramp-up: 10 s, time of ramp-down: 10 s) and the sham protocol were programmed by a dedicated computer software package and saved on the device ahead of the usage. Regular parameters of tDCS were chosen based on pilot study prior to the experiment.

**(b) Functional electrical stimulation therapy**. To deliver the FES therapy, we used the Bio-feedback Neuromuscular Stimulator (MyoNet-BOW-III, NCC Medical Co., LTD, China), a parameter-adjustable transcutaneous stimulator that used self-adhesive surface electrodes. The amplitude of the electric current and the tasks were selected based on patients' needs and adjusted weekly. The motor tasks and the task-related muscles stimulated are given in Table 2. Muscles and nerves were stimulated using symmetrical biphasic current pulses (frequency: 40 Hz, pulse duration: 250 μs, ramp-up time: 2 s, ramp-down time: 2 s). Regular parameters of FES were chosen based on pilot study prior to the experiment. The therapist used a hand switch to trigger the stimulation in accordance with the patients arm motion. The FES therapy was started from the training of shoulder and upper arm muscles as the neuromuscular recovery commonly starts from proximal limbs to distal limbs. At first, the subjects were required to voluntarily perform the specified tasks with their paralyzed arms. The FES therapy was delivered when the subject was unable to complete the task. The assistance of the neuroprotheses was reduced to an appropriate level according to the motor recovery level of the subject and would be eventually removed from the protocol. In each session the subject repeated the same tasks for about 60 times and the whole sessions lasted for up to 1 h. The physiotherapist instructed the movements and provided assistance when it was necessary to make sure that all tasks were executed in a physiological way with the FES stimulation. Patients underwent no other treatments.

**Table 2:** The motor tasks and the task-specific stimulated muscles.

**Task-oriented movements**	**Main goal**	**FES stimulated muscles**
1. Move a thick handle spoon or a jar from the table to mouth.	To train the synergistic activation of affected upper limb flexor muscles and muscle strength.	Anterior deltoid muscle, biceps muscle
2. Return a thick handle spoon or a jar from mouth to the table.	To train the synergistic activation of affected upper limb extensor muscles and muscle strength.	Posterior deltoid muscle, triceps muscle
3. Place the grasped jar on a wooden box with a height at least 15 cm.	To train the flexion function of the shoulder joint.	Anterior deltoid muscle, triceps muscle
4. Slide the jar from the maximum flexion position of elbow toward the lateral direction (requirement: keep the elbow as close to the body as possible).	To train external rotation of shoulder and the muscle strength of wrist extensor and digitorum extensor muscles.	Infraspinatus, teres minor, teres major, extensor carpi radialis longus, extensor carpi radialis brevis and extensor carpi ulnaris muscles
5. Rotate forearm from pronator position to supinator position (initial positon requirement: shoulder with neutral position and elbow with 90° flexion).	To train elbow supination and promote isolated movements.	Biceps, supinator muscle
6. Rotate forearm from supinator position to pronator position (initial positon requirement: shoulder with neutral position and elbow with 90° flexion).	To train elbow pronation and promote isolated movements.	Pronator teres, pronator quadratus
7. Forward open the hand (extend fingers) and release the jar grasped in hand (initial positon requirement: shoulder with neutral position and elbow with 90° flexion)	To train the finger extensor muscles and promote isolated movements.	Extensor digitorum, abductor pollicis brevis, abductor pollicis longus, extensor carpi radialis longus, extensor carpi radialis brevis
8. Grasp or pinch small objects and put them into the box with whole/two fingers.	To train the finger flexor muscles and improve fine motor ability of hand.	Flexor digitorum superficialis, flexor digitorum profundus, flexor pollicis brevis, flexor pollicis longus, musculi opponens pollicis

*FES, functional electrical stimulation. The jar used is a brand new full jar*.

### 2.4. Outcome measures

All the subjects were assessed with behavioral outcome measurement scales before and after the 4 weeks of intervention period. Initial and repeat sEMG (surface electromyogram) and TMS assessments were also performed.

**(a) Behavioral outcome measures**. A proximal shoulder/elbow (0–30) and distal wrist/hand subscore (0–24) of modified upper limb combined Fugl-Meyer assessment (cFMA; excluding coordination, speed and reflexes scores) scores were used to evaluate the motor and sensory impairment of the patients. We excluded (1) coordination and speed and (2) reflexes for following reasons: subjects included into this study had no remaining finger extension and could not touch their noses with the index finger fully extended; reflex scores might be invalid for measurement (Crow and Harmeling-van der Wel, 2008). The secondary behavioral outcome measures included Wolf Motor Function Test (WMFT) (Morris et al., 2001) and Modified Ashworth Scale (MAS) of 6 scores (range, 0–5) (Charalambous, 1987). While MAS was mainly used to assess joint spasticity, cFMA and WMFT were used to measure upper extremity motor ability in different aspects.

**(b) sEMG for muscle activation evaluation**. For sEMG assessment, the subject was seated on a chair with his/her forearm naturally extended toward the ground, and was asked to perform, or to attempt as much as possible to perform the seven movements to the maximal position. Each of the seven movements should be performed within a 5-s period according to the assessment protocol with affected side shoulder, elbow, wrist and metacarpophalangeal joints. These seven movements included shoulder flexion, shoulder abduction, elbow flexion, elbow extension, wrist extension, grasping with fingers and hand opening. Seven wireless electrodes were attached on the agonist muscles that corresponded to the seven movements respectively. These seven muscles were the anterior deltoid, the medial deltoid, the long head of biceps brachii, the lateral head of triceps, the extensor carpi radialis longus, the flexor digitorum superficialis, and the extensor digitorum. To ensure the accuracy of electrode placement, we referred to the Non-Invasive Assessment of Muscles European Community project (SENIAM, the surface electromyography for the non-invasive assessment of muscles) recommendations for sEMG electrode placements (Hermens et al., 1999). The sEMG signals were collected by a commercial myoelectric system (Trigno TM Wireless system, Delsys Inc., 20-450 Hz band pass filter). The sampling frequency was set to 2000 Hz. The skin was cleaned with alcohol to reduce impedance before data acquisition. For each trial, data collected from 0.5 s before movement onset to 2.0 s after movement offset were stored in a computer for off-line processing and subsequently analyzed using Matlab (R2016a, MathWorks Inc., USA).

**(c) TMS evaluation**. TMS was used to measure the corticomotor excitability of the lesioned primary motor cortex (M1). TMS pulses were delivered while the subject seated himself/herself with arms rested on legs. The stimulation site on the scalp that produced the largest and most consistent MEPs was marked as the hot spot for each under-test muscle, respectively. MEPs were regarded to be consistent or stable only if, in every 10 TMS pulses delivered to the scalp, at least 5 pulses could elicit an MEP with an intensity of at least 50 μV. We targeted four muscles, which were abductor pollicis brevis, extensor carpi radialis, extensor digitorium, flexor digitorum superficialis muscles. For subjects whose MEPs could not be elicited in the affected limb, we used the maximal stimulator output with magnetic intensity of 4.2 T. Single pulses were delivered over the lesioned M1 via a figure-of-eight coil held tangentially to the scalp at an angle of 45° to the midline with the handle backward. The coil oriented to induce current flow in a posterior to anterior direction in the underlying tissues. The MEPs of the tested muscles were recorded by use of Ag-AgCl electrodes in a belly-tendon montage utilizing Dantec Keypoint EMG machine (Dantec Medical Inc., Denmark) connected to a laboratory laptop to collect the sEMG signal. The resting motor threshold was defined as the minimal output intensity that could stably elicit an MEP of at least 50 μV at the APB muscle on the affected side. It was determined by slowly increasing the output intensity of TMS device at an interval of 5%, starting from 30% of the maximal output intensity. The corticomotor excitability of lesioned M1 was expressed as the peak-to-peak amplitude value and the latency value of MEP. Additionally, we recorded the resting motor thresholds (RMTs) corresponding to the non-lesioned M1.

### 2.5. Data processing and statistical analysis

The data of the five evaluations were summarized on a personal computer and processed in Matlab (R2016a, MathWorks Inc., USA). In order to avoid the influence of the transition state of motion in sEMG assessment, the sEMG data of the first and the last second in each movement were left out, and only the data in the middle 3 s were processed. The truncated data were filtered by a 4th-order, zero-phase, low-pass Butterworth filter with a cutoff frequency of 10Hz and then centered by median subtraction within each channel in each trial for rectification. After being divided by the standard deviation for normalization, the root mean square (RMS) value was calculated to present muscle activation level.

To ensure the reliability of statistical results, the procedures of the statistical analysis were determined based on the properties of the data. The data of different evaluations were tested for normality of distribution with the Shapiro-Wilk method and for homogeneity of variance with the Levene's test. If they satisfied both priori hypotheses, they would be reported with the mean value and the standard deviations in the form: mean (SD); otherwise, they would be reported with the median value and the interquartile range (IQR) in the form: median (IQR). Two aspects including the within-group difference and the between-group difference were assessed. We investigated the within-group difference, i.e., the effect of time (pre-intervention, post-intervention), by comparing the pre-intervention data with the post-intervention data in terms of each group, respectively. The between-group difference, i.e., the effect of group (Group A, Group B), was investigated by first calculating the difference between the pre-intervention data and the post-intervention data in each group and then comparing the difference of Group A and Group B. If data satisfied both priori hypotheses, an independent sample *t*-test and two paired sample *t*-tests would be performed to assess the between-group difference and the within-group difference, respectively; otherwise, a Mann-Whitney signed rank test and Wilcoxon signed rank tests would be performed. Specially in assessing sEMG data, a two-way ANOVA test with independent measures on group and muscle was performed to investigate the between-group difference. Paired-sample *t*-tests or Wilcoxon signed rank tests would be performed as the *post-hoc* analysis.

All statistics were finished with the statistics software, SPSS (V22, IBM, USA) with the basic level of statistical significance set at *p* < 0.05.

## 3. Results

All subjects went through the complete experiment without dropouts. The feedback from subjects showed that the combined protocol was well tolerated by all subjects without any adverse effect. All data from the 30 subjects were used in the following statistical analysis.

**(a) cFMA**. To assess the within-group difference of the cFMA score, a paired sample *t*-test between the pre-intervention cFMA scores and the post-intervention cFMA scores was conducted for each group. Results showed the cFMA score increased significantly from 16.9 (9.50) to 25.4 (9.19) in Group A [*t*_(14)_ = −5.658, *p* < 0.05] while that in Group B increased from 17.5 (12.4) to 22.1 (13.6) also significantly [*t*_(14)_ = −4.974, *p* < 0.05]. An independent sample *t*-test was performed to indicate the between-group difference between Group A and Group B and showed there was a significant difference between the difference within Group A and within Group B [*t*_(28)_ = 2.223, *p* < 0.05].

**(b) WMFT**. In WMFT, both the functional ability score and the task time were analyzed. Results of paired sample *t*-tests showed the functional ability score of Group A increased from 1.56 (0.374) to 1.83 (0.487) significantly [*t*_(14)_ = −3.746, *p* < 0.05] while there was no significant within-group difference of the functional ability score in Group B [*t*_(14)_ = −0.579, *p* = 0.572]. An independent sample *t*-test found there was a significant between-group difference between Group A and Group B [*t*_(28)_ = 2.152, *p* < 0.05]. In terms of the task time, a significant within-group difference was only found in Group A as assessed by the Wilcoxon signed rank test (*Z* = −2.197, *p* < 0.05). There was a significant between-group difference between Group A and Group B as assessed by the Mann-Whitney signed rank test (*Z* = 1.728, *p* < 0.05).

**(c) MAS**. The results of paired sample *t*-tests revealed there was a significant within-group difference on the MAS scores in Group A [*t*_(14)_ = 5.236, *p* < 0.05] and Group B [*t*_(14)_ = 2.739, *p* < 0.05]. An independent sample *t*-test was performed to assess the between-group difference and found there was no significant difference between the difference within Group A and within Group B [*t*_(28)_ = −1.813, *p* = 0.081]. A further analysis with paired sample *t*-tests was performed on the subscores of different joints in the two groups. Similar results in Group A and Group B were obtained that there was a significant within-group differences in the wrist joint (*Z*_*A*_ = −2.825, *p*_*A*_ < 0.05; *Z*_*B*_ = −2.264, *p*_*B*_ < 0.05) and in the metacarpophalangeal joints (*Z*_*A*_ = −2.931, *p*_*A*_ < 0.05; *Z*_*B*_ = −2.264, *p*_*B*_ < 0.05) but not in the elbow joint (*Z*_*A*_ = −1.912, *p*_*A*_ = 0.056; *Z*_*B*_ = 0.000, *p*_*B*_ = 1.000).

**Figure 3:**
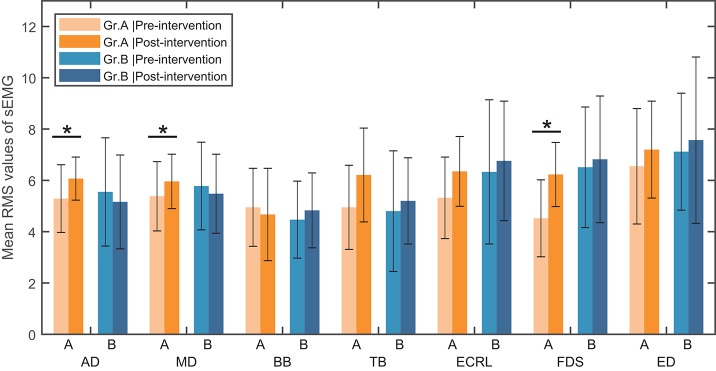
Mean RMS value of sEMG signal in seven muscles and standard deviation across each group (Group A or Group B). AD, anterior deltoid; MD, middle deltoid; BB, biceps brachii; TB, triceps brachii; ECRL, extensor carpi radialis longus; FDS, flexor digitorum superficialis; ED, extensor digitorum; Gr.A, Group A; Gr.B, Group B. ^*^Significance level *P* < 0.05.

**(d) sEMG assessment for activation of the muscles**. The seven upper limb muscles were assessed with the muscle activation level in the paralyzed arm. In each movement, only the sEMG data of the most activated muscle were analyzed. A two-way ANOVA test was performed on the muscle activation level with independent measures on group and muscle. Results showed that there were no interaction between group and muscle [*F*_(6, 196)_ = 0.918, *p* = 0.483] and no significant main effect of muscle [*F*_(6, 196)_ = 0.540, *p* = 0.778]. However, there was a significant main effect of group [*F*_(1, 196)_ = 7.483, *p* < 0.05]. The *post-hoc* analysis in Group A showed that there was a significant within-group difference of the muscle activation level in the anterior deltoid (*Z* = −2.045, *p* < 0.05), the extensor carpi radialis longus [*t*_(14)_ = −2.191, *p* < 0.05] and the flexor digitorum superficialis [*t*_(14)_ = −3.650, *p* < 0.05], respectively (see Figure 3). No significant within-group difference in the muscle activation level was found in Group B.

**(e) TMS evaluation**. Paired sample *t*-tests were applied on RMTs of non-lesioned M1. The results showed that, while there was a significant within-group increase from 48.3 (2.53) % to 53.3 (2.81) % in Group A [*t*_(14)_ = −3.570, *p* < 0.05], there was no significant within-group difference in Group B [*t*_(14)_ = −0.646, *p* = 0.528]. An independent sample *t*-test revealed there was a significant between-group difference between Group A and Group B [*t*_(28)_ = 3.258, *p* < 0.05]. As for the lesioned M1, due to the fact that MEPs could not be consistently elicited, no statistical analysis was performed. Tables 3, 4 present the peak-to-peak amplitude and the latency of MEPs corresponding to the four muscles concerned in Group A and Group B, respectively. In both Tables 3, 4, the bold text was used to indicate an increased amplitude while the bold and italic text was used to indicate a decreased latency in the post-intervention MEPs. It can be observed that there were more cases with an increased amplitude and more cases with a decreased latency in Group A (*N*_*bold*_ = 30, *N*_*bold*&*italic*_ = 33) than in Group B (*N*_*bold*_ = 25, *N*_*bold*&*italic*_ = 24).

**Table 3:** Results of Motor evoked potentials (MEPs) on the affected side in Group A.

**Subject**	**Amplitude of MEPs (mV)**				**Latency of MEPs (ms)**			
	**APB**	**FDS**	**ED**	**ECRL**	**APB**	**FDS**	**ED**	**ECRL**
	**GROUP A – PRE-INTERVENTION**							
1	0.436	0.195	0.354	0.249	27.38	28.87	27.12	26.57
2	0.199	0.0716	0.0296	0.1033	26.6	26.644	23.011	25.111
3	–	–	–	–	–	–	–	–
4	–	–	–	–	–	–	–	–
5	–	–	–	–	–	–	–	–
6	–	–	–	–	–	–	–	–
7	0.028	0.0319	0.026	0.024	32.49	29.05	27.83	31.31
8	–	–	–	–	–	–	–	–
9	–	–	–	–	–	–	–	–
10	–	–	–	–	–	–	–	–
11	0.162	–	0.042	–	28.823	–	28	–
12	–	–	–	–	–	–	–	–
13	–	–	–	–	–	–	–	–
14	0.147	0.088	0.053	0.081	32.55	24.23	30.8	23.4
15	0.034	–	–	0.025	22.54	–	–	–
	**GROUP A – POST-INTERVENTION**							
1	0.395	**0.275**	0.314	**0.312**	***25.08***	***24.1***	***24.93***	***24.58***
2	0.137	0.032	**0.032**	0.089	27.37	***25.7***	23.74	***23.08***
3	**0.053**	**0.012**	**0.022**	**0.027**	31.17	***28.74***	***24.28***	***27.122***
4	**0.023**	–	–	–	***24.25***	–	–	–
5	**0.035**	–	**0.087**	**0.111**	***30.38***	–	***29.9***	***24.68***
6	**0.072**	**0.023**	–	–	***35.1***	***33.81***	–	–
7	**0.029**	0.031	**0.04**	0.021	***30.69***	***27.02***	28.025	***30.26***
8	**0.438**	**0.383**	**1.358**	**1.7**	***24.13***	***22.61***	***21.16***	***20.16***
9	–	–	–	–	–	–	–	–
10	**0.213**	–	–	–	***30.45***	–	–	–
11	**0.222**	**0.039**	**0.021**	**0.042**	***26.22***	***28.057***	29	***27.14***
12	**0.050**	–	–	–	***36.47***	–	–	–
13	**0.075**	**0.028**	–	–	***26.7***	***25.6***	–	–
14	**0.172**	0.074	**0.075**	0.077	***28.49***	***21.2***	***21.13***	***21.96***
15	**0.049**	–	–	–	31.21	–	–	–

*Data in bold but not italic text indicate an increase in magnitude after intervention.

*Data in bold and italic text indicate a decrease in magnitude after intervention.

*APB, abductor pollicis brevis; FDS, flexor digitorum superficialis; ED, extensor digitorum; ECRL, extensor carpi ralialis longus*.

**Table 4:** Results of Motor evoked potentials (MEPs) on the affected side in Group B.

**Subject**	**Amplitude of MEPs (mV)**				**Latency of MEPs (ms)**			
	**APB**	**FDS**	**ED**	**ECRL**	**APB**	**FDS**	**ED**	**ECRL**
	**GROUP B – PRE-INTERVENTION**							
1	0.1	0.012	0.011	0.01	25.84	22.63	25.65	22.42
2	–	–	–	–	–	–	–	–
3	0.059	0.1	0.087	0.075	29.72	21.19	22.05	21.8
4	–	–	–	–	–	–	–	–
5	0.031	0.031	0.053	0.034	34.81	29.56	32.32	30.06
6	0.16	0.045	0.049	0.043	26.58	25.81	20.84	25.34
7	–	–	–	–	–	–	–	–
8	0.061	0.049	0.048	0.043	20.13	22.82	20.7	20.75
9	–	0.067	0.067	–	–	29.43	30.35	–
10	–	–	–	–	–	–	–	–
11	0.026	0.019	0.048	–	33.44	31.2	31.72	–
12	–	–	–	–	–	–	–	–
13	0.022	–	–	0.022	21.24	–	–	30.14
14	–	–	–	–	–	–	–	–
15	–	–	–	–	–	–	–	–
	**GROUP B – POST-INTERVENTION**							
1	**0.12**	0.011	**0.015**	0.01	***26.9***	***21.3***	26.7	***22.2***
2	–	–	–	–	–	–	–	–
3	**0.152**	**0.123**	**0.098**	**0.118**	31.25	***20.47***	***20.33***	***21.55***
4	–	–	–	–	–	–	–	–
5	**0.062**	**0.122**	**0.124**	**0.098**	***29.96***	***28.19***	***28.42***	***25.69***
6	0.16	**0.069**	**0.07**	0.037	27.07	***20.49***	21.99	***20.95***
7	–	–	–	–	–	–	–	–
8	0.024	**0.059**	**0.065**	**0.065**	31.93	***20.93***	20.7	21.01
9	–	–	0.041	–	–	–	***26***	–
10	**0.387**	**0.037**	**0.243**	**0.060**	***28.08***	***24.36***	***28.93***	***28.78***
11	0.021	**0.0392**	**0.0513**	**0.028**	***31.66***	***29.61***	31.74	***26.95***
12	–	–	–	–	–	–	–	–
13	**0.024**	–	–	0.0216	***21.09***	–	–	30.16
14	**0.025**	**0.013**	–	–	***30.84***	***32.8***	–	–
15	–	–	–	–	–	–	–	–

*Data in bold but not italic text indicate an increase in magnitude after intervention.

*Data in bold and italic text indicate a decrease in magnitude after intervention.

*APB, abductor pollicis brevis; FDS, flexor digitorum superficialis; ED, extensor digitorum; ECRL, extensor carpi ralialis longus*.

## 4. Discussion

In this research, a novel clinical protocol that included applying bilateral tDCS as an add-on treatment prior to FES therapy was investigated. The efficacy of the proposed protocol that consisted of 20 min of bilateral tDCS intervention followed by 1 h of FES therapy was examined over the course of a 4-week rehabilitation program. A randomized controlled experiment was conducted on 30 qualified subjects with severe chronic stroke. Five assessments including 3 behavioral outcome measurement scales (cFMA, WMFT, MAS), the sEMG evaluation and the TMS assessment were performed to compare between the proposed protocol with active tDCS+FES and the control group with sham tDCS+FES. The WHOQOL-BREF scale was not used because it has little relation with motor function assessment (Skevington et al., 2004). As an alternative, we collected oral feedback from subjects and found the proposed protocol was well tolerated by all subjects without any adverse effect.

The statistical results of the cFMA scores revealed that, although both the proposed protocol and the protocol with FES alone improved the upper extremity motor ability in stroke patients, the proposed protocol was found to be more capable than the protocol with FES alone. The secondary outcome measure of WMFT showed that improvements in upper extremity motor abilities were only found in the group with the proposed protocol but not in the control group. Thus, in terms of WMFT, the proposed protocol was more efficient in improving upper extremity motor abilities than the protocol with FES alone. The results of the MAS measure indicated both of the protocols were able to elicit an overall reduction in upper extremity spasticity. However, there was no significant difference between the two protocols in reducing spasticity. Since the regular therapy of FES was included in both of the protocols, we assume the reduction of upper extremity spasticity was caused by FES therapy alone.

In sEMG assessment, results showed that, while no improvement of muscle activation level was found with the protocol of FES alone, the proposed protocol was able to improve the muscle activation level in 3 muscles of the paralyzed arm: the anterior deltoid, the extensor carpi radialis longus, and the flexor digitorum superficialis. In addition, the results of the 2-way ANOVA test indicated the proposed protocol is superior to the protocol with FES alone at improving muscle activation levels overall. In the TMS evaluation, although no statistical analysis was performed on the lesioned side, more cases with an increased amplitude and more cases with a decreased latency was found in the group with the proposed protocol, indicating that the proposed protocol was more capable of increasing the corticomotor excitability in the lesioned M1. Thus, we conclude that the proposed protocol can facilitate improvements in upper extremity motor abilities in severe chronic stroke patients and is more beneficial than the protocol with FES therapy alone.

It is worth noting that the tDCS treatment with a bilateral montage was incorporated in the proposed protocol. The bilateral montage was chosen in an effort to improve stroke by balancing the corticomotor excitability while unilateral montages mostly affect only one side of the hemisphere. The efficacy of the bilateral montage over stroke patients has previously been investigated by Mahmoudi et al. (2011) who demonstrated a positive effect of the bilateral montage by comparing it with several other tDCS montages (Mahmoudi et al., 2011). In MEP assessment, we found an inhibition of the cortical excitability in the non-lesioned M1 and a facilitation of the cortical excitability in the lesioned M1, which were consistent with the rationale of the bilateral montage in intracortical inhibition (Sehm et al., 2013). Therefore, our results may suggest an underlying approach of improving the upper extremity motor abilities in stroke by balancing the cortical excitability.

In carrying out the protocol, we chose to apply tDCS as an add-on treatment prior to the FES therapy, rather than apply both therapies simultaneously. This was determined based on the following considerations: (1) previous research showed corticomotor excitability increased approximately 150% above baseline for up to 90 min after the end of tDCS intervention as revealed by transcranial magnetic stimulation on healthy subjects (Nitsche and Paulus, 2001), which means our 60 min of FES therapy might be within the post-tDCS effect time; (2) the concurrent intervention of tDCS and FES therapy might activate the homeostatic mechanism and reduce the treatment effect as the homeostatic mechanism acts to prevent excessive increases in excitability in cortical networks and maintain its stability (Abraham and Bear, 1996).

The homeostatic mechanism refers to the theory that, when the cortical excitability has already been elevated, a long-term-potentiation-like increase in synaptic efficacy or a raise in cortical excitability may lead to long-term-depression-like decreases and the downregulation of the cortical excitability (Nitsche et al., 2007). It has been demonstrated in some related studies, such as by Nitsche et al. (2007) who demonstrated a reduction in corticomotor excitability with concurrent application of paired associative stimulation (PAS) and anodal tDCS (Nitsche et al., 2007). The study by Schabrun et al. (2013) showed that concurrent application of neuromuscular electrical stimulation (NMES) and anodal tDCS failed to induce summative effects on corticomotor excitability of healthy subjects (Schabrun et al., 2013). Another study by Rosenkranz et al. (2000) revealed tDCS intervention during motor training can diminish learning effect due to an interaction of rapid training induced plasticity and the tDCS (Rosenkranz et al., 2000).

However, based on our results, the effect of the FES therapy was not reduced by combining the bilateral tDCS therapy. Moreover, there was a significant increase in the motor functions of the paretic arm compared to the condition with the FES therapy alone. Thus, our results suggest a potential approach of evading the homeostatic mechanism by asynchronous stimulation. We assume the cortical activation during assisted FES motor training may not interfere directly with post-tDCS excitability shifts. In other words, the potential comprehensive modification of cortical GABAergic inhibitory networks resulting from our protocol may be mutually complementary rather than antagonistic.

To our knowledge, this is the first randomized controlled trial that investigated the long-term efficacy of applying tDCS as an add-on treatment prior to traditional FES therapy. Based on our results, we conclude that the proposed protocol can facilitate improvements in upper extremity motor abilities in severe chronic stroke patients and is more beneficial than the protocol with FES therapy alone. The feedback from subjects show the proposed protocol combining the tDCS and the FES therapy can be well tolerated. Our results suggest new trials with combined intervention in both the central nervous system and the peripheral nervous system. In terms of limitations, since only stroke patients with unilateral arm paralysis were recruited and tested, more types and cases of stroke are needed. In addition, due to the demographics of the patients that were available for the study, both groups were skewed toward males. To further investigate in the interaction between the tDCS treatment and the FES therapy in the protocol, more clinical trials with various intervention conditions and more diverse demographics are also needed.

## Author contributions

NS and BZ contributed in carrying out the experiment, data analysis, and drafting the manuscript. JJ contributed in coordinating process of patient recruitment and participation. DZ conceived of the study and gave instructions on experimental design.

### Conflict of interest statement

The authors declare that the research was conducted in the absence of any commercial or financial relationships that could be construed as a potential conflict of interest.
